# Correlation between acellular dermal matrix (ADM) volume and breast implant size selection among patients undergoing prepectoral direct-to-implant (DTI) breast reconstruction using complete ADM

**DOI:** 10.1097/MD.0000000000031344

**Published:** 2022-11-18

**Authors:** Yoon Soo Kim, Ho Sung Kim, Seok Kyung In, Byeong Seok Kim, Hyo Young Kim, Hong Il Kim, Hyung Suk Yi, Jin Hyung Park

**Affiliations:** a Department of Plastic and Reconstructive Surgery, Kosin University College of Medicine Busan, Busan, Republic of Korea.

**Keywords:** acellular dermal matrix, breast, implant

## Abstract

In prepectoral direct-to-implant breast reconstruction using the acellular dermal matrix (ADM)-assisted technique, breast volume asymmetry can occur. We hypothesized that ADM size influences implant size selection in prepectoral breast reconstruction with implant coverage using an ADM. We investigated factors influencing implant size selection and provide guidelines for surgeons.

**Methods:**

A retrospective chart review was performed to identify all patients who underwent prepectoral direct-to-implant breast reconstruction between January 2017 and October 2020. We assessed patient characteristics, preoperative expected implant volume, ADM size, volume of implant used in surgery, and symmetry scale of aesthetic item scale (AIS) 6 months after surgery. We compared the symmetry score of AIS between a group in which the preoperative expected silicone implant size (ES) was used and a group in which a silicone implant of a smaller size than planned (SS) was used.

**Results:**

Patient characteristics, including age, body mass index (BMI), and excised breast volume, were similar between the groups (*P* > .05). ADM size had a significant effect on implant size selection (odds ratio = 1.760, *P* < .01). The symmetry score of AIS was higher in the SS group.

**Conclusions:**

ADM size must be considered when selecting implant size in prepectoral direct-to-implant breast reconstruction using the ADM-assisted technique.

## 1. Introduction

Immediate implant-based reconstruction is the most common approach for correcting postmastectomy defects and restoring the breast mound.^[1,2]^ For the past decade, implant-based breast reconstruction methods have evolved from 2-stage reconstruction, with tissue expander insertion followed by expansion and implant exchange, to direct-to-implant breast reconstruction. Whether performed as a staged or single operation, dual-plane reconstruction has become the most widely used technique. In this technique, the implant is secured along the inferolateral pole using biomaterial adjuncts such as an acellular dermal matrix (ADM) or surgical mesh.^[3–6]^

However, over recent years, the rate of prepectoral implant-based breast reconstruction has risen because of the use of several devices.^[7]^ In particular, ADM is widely used because it provides structural support and forms the implant pocket, while also reducing the rate of capsular contracture in prepectoral breast reconstruction.^[8–10]^

Despite the extensive literature on prepectoral breast reconstruction, debate persists regarding techniques that involve covering the silicone implant with ADM and inserting it into the prepectoral space. The present study on the use of ADM for prepectoral implant-based breast reconstructions differs from previous studies in terms of the method of ADM coverage used and the size of the ADM used.^[11–13]^

We have experienced postoperative breast asymmetry in prepectoral direct-to-implant breast reconstruction using the ADM-assisted technique, even when there were no problems with the surgical procedure overall. Most of the asymmetry occurred in the breast on the reconstructed side, as it had a larger volume than that in the unaffected breast. We hypothesized that the ADM volume used in the prepectoral direct-to-implant breast reconstruction using the ADM-assisted technique influences postoperative breast volume and, therefore, symmetry. We reasoned that postoperative breast symmetry would be improved if the silicone implants used were smaller than what was planned during surgery, as the volume of the ADM used would increase the total volume and contribute to the asymmetry postoperatively.

The purpose of the present study was to investigate factors influencing implant size selection and to provide guidelines for plastic surgeons on choosing a suitably sized implant in prepectoral direct-to-implant breast reconstruction using the ADM-assisted technique.

## 2. Materials and Methods

### 2.1. Patient selection

We performed a retrospective review of all patients with breast cancer who underwent nipple-sparing mastectomy (NSM) using inframammary fold (IMF) incision and prepectoral direct-to-implant breast reconstruction with the complete coverage technique using ADM at our institution between January 1, 2017 and October 31, 2020. This study was approved by the Institutional Review Board of Kosin University Gospel Hospital (KUGH-2020-09-013). Written informed consent was obtained from all patients, and the study was conducted in accordance with the principles of the Helsinki Declaration.

Appropriate patient selection is vital to ensure a good outcome in prepectoral direct-to-implant breast reconstruction using the ADM-assisted technique. We selected patients in accordance with the Joint Guidelines of the Association of Breast Surgeons and the British Association of Plastic, Reconstruction, and Aesthetic Surgeons.^[14]^

Two groups of patients who underwent prepectoral direct-to-implant breast reconstruction using the ADM-assisted technique were evaluated: those who received a silicone implant of a smaller size than planned (SS) and those who received the preoperative expected silicone implant size (ES). The ES group comprised patients who underwent the procedure between January 2017 and June 2018, while the SS group comprised those who were treated between June 2018 and October 2020. The same surgeons operated on both cohorts, thereby minimizing surgeon-related variables. Patients who were current smokers, had uncontrolled diabetes mellitus, had severely ptotic breasts, underwent preoperative and postoperative radiotherapy, and underwent bilateral breast reconstruction were thought to be bias in this study to investigate the factor effect on postoperative breast volume, therefore, excluded from the study.

### 2.2. Surgical techniques

**Figure 1.**
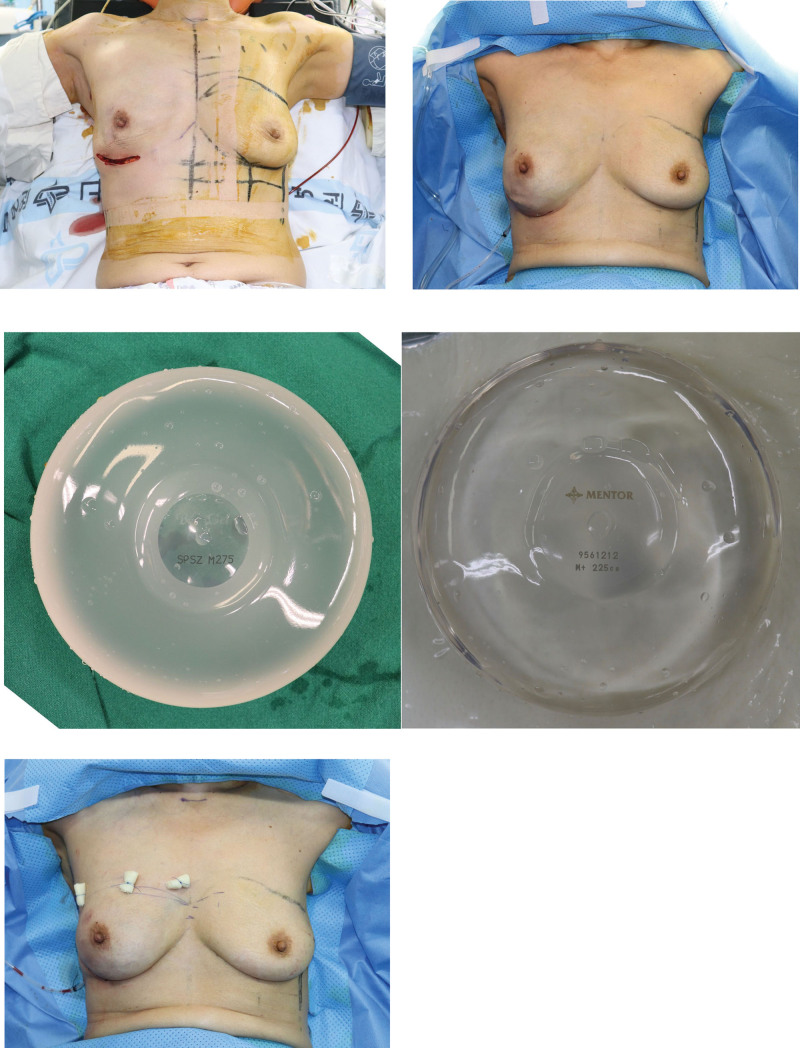
Prepectoral direct-to-implant breast reconstruction with ADM assisted technique. (a) Postmastectomy state. (b) Intraoperative sitting position photograph showing proper symmetry of breast using an implant sizer. (c) Breast implant covered with ADM. At this stage, ES group involves selecting the silicone implant of the same size as implant sizer. On the other hand, SS group selected a silicone implant with a size of 25 cc (SS_25_) or 50 cc (SS_50_) smaller that the implant sizer. (d) Immediate postoperative photograph showing 3 bolster sutures in the breast upper pole. ADM = acellular dermal matrix, ES = group in which the preoperative expected silicone implant size, SS = group in which a silicone implant of a smaller size than planned.

The IMF incision was considered the preferred procedure, unless there was preoperative evidence of the need for areolar incisions due to the location and size of the underlying cancer. Single-stage reconstruction was performed in all patients who had undergone NSM. A single plastic surgeon performed the reconstructive procedures in both cohorts, using standardized operative techniques. Following mastectomy, the most appropriate implant was selected intraoperatively using an implant sizer to ensure breast symmetry. In the ES group, a silicone implant the same size as the implant sizer was selected. In contrast, in the SS group, a silicone implant with a size 25 cc (SS_25_ group) or 50 cc (SS_50_ group) smaller than the implant sizer was selected, to accommodate for the ADM size and assist in obtaining a better result for postoperative breast symmetry (Fig. 1).

**Figure 2.**
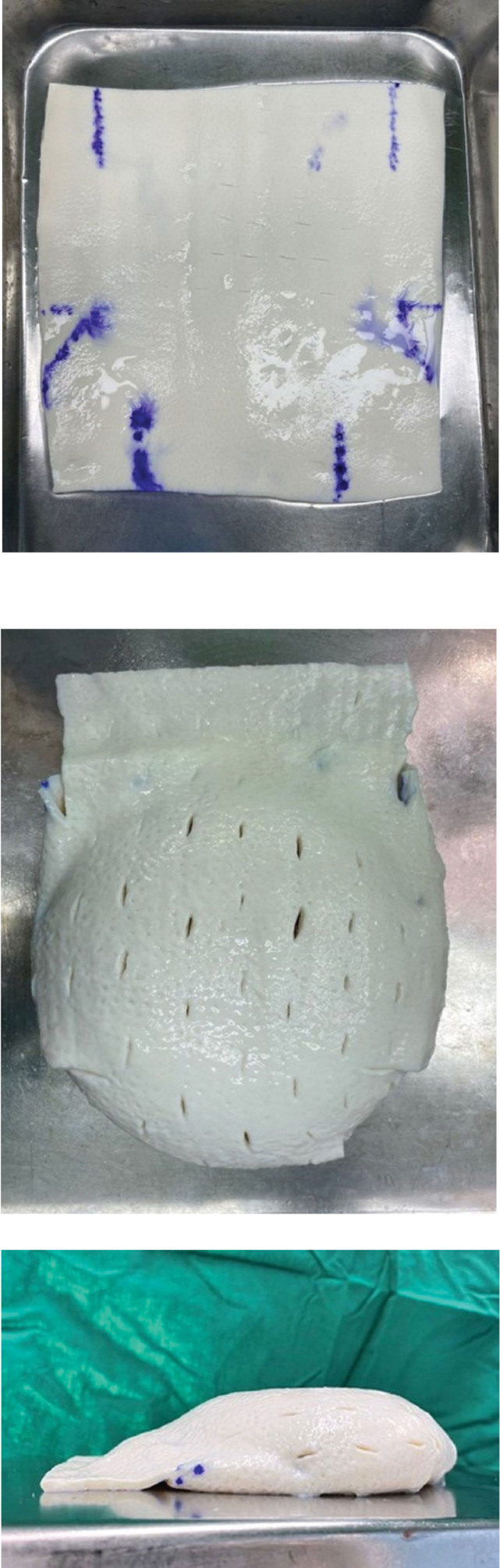
Clinical photographs of the complete coverage technique using ADM. (a) Design of coverage technique. The side of the ADM’s dermis was divided into 3 equal parts, one on the back and the others on the bottom, spreading out upward. Triangles were drawn on both sides of the point where the extension of the width met with the lower one-third point. The point of the extension of the implant width at the top was marked at the bottom and half of the point of the lateral, which was connected to the area where the implant widths met each other, creating triangles on both sides. The solid line part of the ADM was cut off to prevent unnecessary overlapping. (b) Clinical photograph of frontal view after complete coverage. Seroma prevention was achieved with the addition of a stab incision using the scalpel blade 11 times to the ADM. (c) Clinical photograph of lateral view after complete coverage. The remaining cut ADM pieces in the middle of the ADM coverage design were used to remove the upper aspect’s coverage from the blank portion of the implant’s posterior aspect. ADM = acellular dermal matrix.

The pectoralis major muscle was left intact, and the wounds were irrigated with gentamicin. The ADMs used in our series were the MegaDerm (L&C Bio, Seoul, Republic of Korea) and the CG CryoDerm (CGBio Corp., Seongnam, Republic of Korea). Highly cohesive, round, smooth, silicone gel-filled implants were used (BellaGel SmoothFine^®^; Hans Biomed, Seoul, Republic of Korea). Seroma prevention was achieved with the addition of a stab incision using the scalpel blade 11 times to the ADM. The side of the ADM’s dermis was divided into 3 equal parts, one on the back and the others on the bottom, spreading out upward. The implant was placed in the middle of the ADM where the implant was flipped, and an extension of the width of the implant was drawn up and down. Triangles were drawn on both sides of the point where the extension of the width met with the lower one-third point. The point of the extension of the implant width at the top was marked at the bottom and half of the point of the lateral, which was connected to the area where the implant widths met each other, creating triangles on both sides. The solid line part of the ADM was cut off to prevent unnecessary overlapping. The lower pole pocket of the suture was created using 3-pronged ADM Vicryl® 3-0 sutures (Ethicon Inc., Somerville, NJ) located at the bottom of the implant. The coverage on the side of the implant was fixed to the lower pocket of the lateral area on both sides of the ADM. The part in the upper corner of the ADM was fixed to the center of the implant, completing the coverage of the implant’s upper lateral aspect. Lastly, the remaining cut ADM pieces in the middle of the ADM coverage design were used to remove the upper aspect’s coverage from the blank portion of the implant’s posterior aspect (Fig. 2).

A closed-suction drain was used in each group. Next, the skin flaps were closed in a standard fashion. After wound dressing, all patients were provided with an elastic bandage and a surgical compression post-surgery bra. The drain was removed when the output was <30 mL over a 24 hours period, which usually occurred on postoperative days 10 to 14. The drainage protocol was the same in both cohorts.

### 2.3. Evaluation of volume symmetry scale

**Figure 3.**
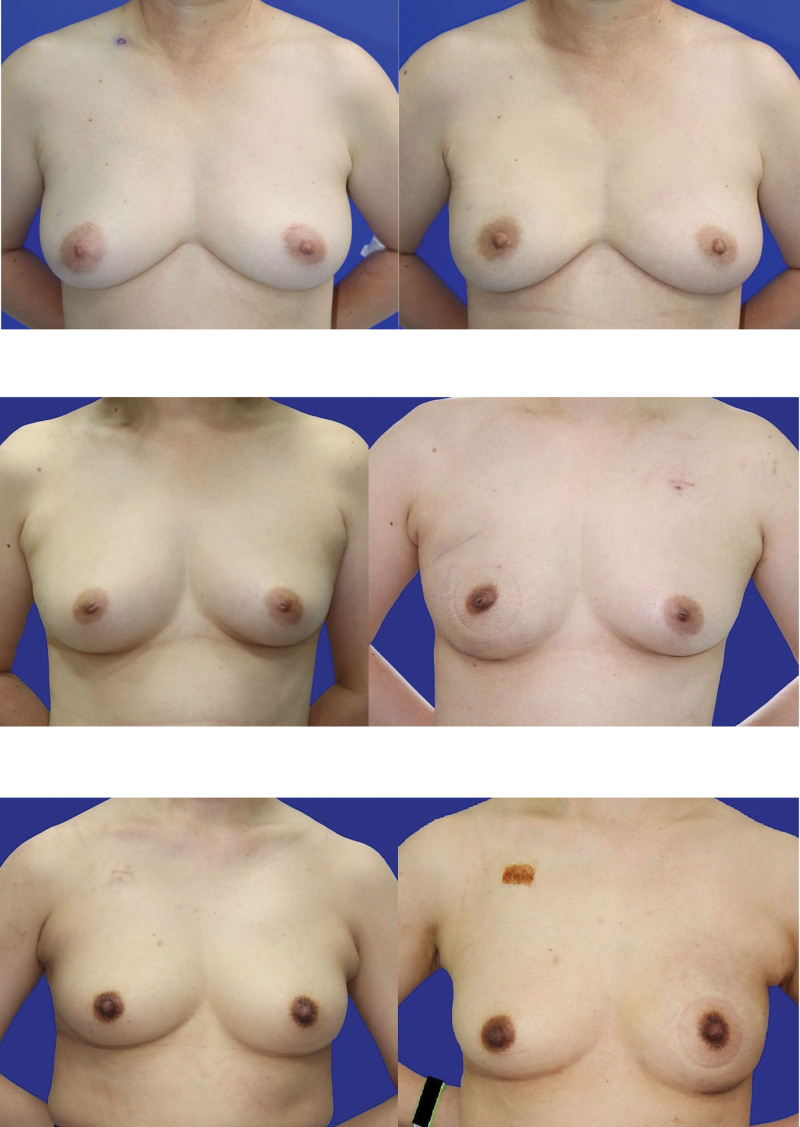
Representative photographs (frontal view) for evaluated the breast symmetry. (a) Representative pre-postoperative image of SS_50_ taken at the 6-month-follow-up. 30-year-old woman who underwent left NSM and prepectoral direct-to-implant breast reconstruction with ADM assisted technique. (Implant sizer; 300cc, Breast silicone implant 250cc) An excellent result score as 4. (b) Representative pre-postoperative image of SS_25_ taken at the 6-month-follow-up. 30-year-old woman who underwent left NSM and prepectoral direct-to-implant breast reconstruction with ADM assisted technique. (Implant sizer; 300cc, Breast silicone implant 275cc) A good result score as 3.5. (c) Representative pre-postoperative image of ES taken at the 6-month-follow-up. 30-year-old woman who underwent left NSM and prepectoral direct-to-implant breast reconstruction with ADM assisted technique. (Implant sizer; 300cc, Breast silicone implant 300cc) A fair result score as 3. ADM = acellular dermal matrix, NSM = nipple-sparing mastectomy.

We evaluated breast symmetry according to the aesthetic items scale (AIS). AIS is the tool that the postoperative aesthetic outcome of breast reconstruction was evaluated for, and Visser et al introduced a method for scoring aesthetic outcome after breast reconstruction with the use of 5 standardized photographs, which are then rated using a 5-point Likert scale with respect to volume, shape, symmetry, scars, and nipple areola complex: “1 = very dissatisfied,” “2 = dissatisfied,” “3 = neutral,” “4 = satisfied,” and “5 = very satisfied.”^[15,16]^ To assign AIS, all of the participants took clinical photographs with standing position under the same conditions. These photographs did not feature the individuals’ faces or any other personal information in the anterior, oblique and lateral view. In addition, 2 plastic surgeons who were not involved in the operation independently assessed AIS using clinical photographs at the 6-month follow-up. These photographs did not include any personal information about the participants. Two plastic surgeons who were not involved in the surgery evaluated the clinical photographs taken at the 6month follow-up. The means of the 2 scores were then calculated^[16]^ (Fig. 3).

### 2.4. Statistical analysis

The collected data were analyzed using SPSS Win ver. 18.0 (SPSS, Inc., Chicago, IL). In all statistical comparisons, a *P* value of ≤.05 was considered significant. Data analysis was performed as follows:

Frequency analysis was conducted on the baseline patient characteristics, including age, body mass index (BMI), history of diabetes mellitus, excised mass weight, expected silicone implant size, actual size of silicone implant used in surgery, ADM size, ADM type, and chemotherapy.

Logistic regression analysis was conducted to identify the effects of age, BMI, diabetes mellitus, excised mass weight, ADM size, ADM type, and chemotherapy on silicone implant size selection.

An independent-sample *t* test was conducted to verify that there were significant differences in the volume symmetry scale between the 2 groups (ES and SS).

One-way analysis of variance (ANOVA) was conducted to verify whether the symmetry score of AIS differed significantly among the 3 groups (ES, SS_25_, and SS_50_).

## 3. Results

During the study period, a total of 119 patients underwent prepectoral direct-to-implant breast reconstruction using the ADM-assisted technique (ES group, n = 62 [51.7%]; SS group, n = 57 [48.3%]). All patients underwent unilateral single-stage, direct-to-implant breast reconstruction after mastectomy. The patient characteristics, including age, BMI, excised breast volume, silicone implant size, and average ADM volume, were similar between the groups and are listed in Table 1. Preoperative chemotherapy was performed in 25 patients in the ES group and in 28 patients in the SS group. Postoperative chemotherapy was performed in 10 and 4 patients, respectively (Table 1).

**Table 1:** Patient characteristics.

Characteristic	Implant selection type		
	ES	SS	*P*
No. of patients (%)	62 (51.7%)	57 (48.3%)	
Age, yrs	48.98 ± 7.76	47.56 ± 9.22	.141
BMI, kg/m^2^	22.79 ± 2.76	23.74 ± 3.30	.143
Diabetes mellitus	2	3	
Excised mass weight, g	241.65 ± 114.96	278.07 ± 150.51	.020
Silicone implant size Expected silicone implant size (cc) Actual size of silicone implant used in surgery (cc)	261.94 ± 83.75 261.94 ± 83.75	339.74 ± 103.20 300.44 ± 98.08	.105 .217
ADM size, cm^2^			
6 × 12	0	1	
6 × 16	2	0	
7 × 17	11	4	
7 × 18	13	12	
8 × 16	25	13	
8 × 18	9	15	
16 × 16	2	12	
Average ADM size, cm^2^	130.24	155.02	.354
ADM type			
MegaDerm	16	12	
CryoDerm	46	45	
Chemotherapy			
Preoperative	25	28	
Postoperative	10	4	
None	27	25	

ADM = acellular dermal matrix, BMI = body mass index, ES = estimated size used group, SS = smaller than sized sizer used group.

The noteworthy results from the logistic regression analysis are listed in Table 2. Logistic regression analysis was conducted to identify the effects of age, BMI, diabetes mellitus, excised mass weight, ADM size, ADM type, and chemotherapy on silicone implant size selection. The logistic regression model was statistically significant (Hosmer and Lemeshow *χ*² = 13.998, *P* = .82), and the explanatory power of the regression model was approximately 17.5% (Nagelkerke *R^2^* = 0.175). The significance verification of the regression coefficients showed that ADM size had a significant effect on silicone implant size selection (odds ratio = 1.760, *P* < .01). Thus, it was assessed that a single-step increase in ADM size would increase the likelihood of selecting a smaller-than-planned silicone implant by approximately 1.760 times. In contrast, age, BMI, diabetes mellitus, weight of excised mass, ADM type, and chemotherapy did not have a significant effect on silicone implant size selection (Table 2).

**Table 2:** Factors affecting implant volume selection.

Dependent variable	Independent variable	B	S.E.	OR	95% CI	*P*
Silicone implant size selection	Age	-0.030	0.026	0.970	0.921–1.021	.247
	BMI	0.126	0.085	1.134	0.960–1.339	0.139
	Diabetes mellitus	-0.348	1.020	0.706	0.096–5.217	.733
	Excised mass weight	0.000	0.002	1.000	0.996–1.003	.826
	ADM size	0.565	0.193	1.760*	1.204–2.571	.003*
	ADM type	0.295	0.271	1.344	0.789-2.287	0.277
	Chemotherapy	0.054	0.227	1.055	0.677–1.646	.812
-2LL: 146.801, Nagelkerke *R*^2^ = 0.175, Hosmer & Lemeshow test: *χ*²=13.998 (*P* = .82)						

ADM = acellular dermal matrix, BMI = body mass index.

* *P* < .05.

It was determined, via the use of an independent-sample t-test, whether or not there was an item that demonstrated a statistically significant difference between the ES group and the SS group. As a result, only the symmetry score of AIS differed significantly between the 2 groups (*t* = 7.528, *P* < .05), and that the symmetry score of AIS was higher in the SS group (M = 3.96) than in the ES group (M = 2.76). In other words, this indicates that the SS group was more symmetric than the ES group (Table 3).

**Table 3:** Correlation between aesthetic item scale and breast implant size selection.

Dependent variable	Group	Number of samples	Mean	Standard deviation	t	*P*
Volume	ES	62	4.20	0.76	0.373	.710
	SS	57	4.15	0.93		
Shape	ES	62	3.91	0.96	−.0512	.609
	SS	57	3.99	0.88		
Symmetry	ES	62	2.76	0.95	7.528*	.000
	SS	57	3.96	0.78		
Scars	ES	62	4.09	1.04	−0.764	.45
	SS	57	4.22	.82		
Nipple areolar complex	ES	62	4.16	0.74	0.57	.954
	SS	57	4.15	0.81		

ES = estimated size used group, SS = smaller than sized sizer used group.

* *P* < .05.

One-way ANOVA was conducted to verify whether the symmetry score of AIS differed significantly among the 3 groups (ES, SS_25_, and SS_50_), and a significant difference was found (F = 35.902, *P* < .05). Scheffe’s post hoc analysis for symmetry score of AIS showed that the score was higher in the SS_50_ group than in the SS_25_ group (Table 4).

**Table 4:** Breast volume symmetry scale comparison between 3 groups.

Dependent variable	Group	Number of samples	Mean	Standard deviation	F	*P*	Scheffe’s
Volume symmetry scale	ES (a)	62	2.76	0.953	35.902*	.000	a < b < c
	SS_25_ (b)	24	3.54	0.779			
	SS_50_(c)	33	4.27	0.626			

ES = estimated size used group, SS25 = 25cc smaller than sized sizer used group, SS50 = 50cc smaller than sized sizer used group.

* *P* < .05.

## 4. Discussion

The present study indicated that using a silicone implant a size smaller than planned confers better postoperative breast symmetry than choosing a silicone implant with the same size as the implant sizer during surgery. In addition, the results showed that postoperative breast symmetry was highest when a size 50 cc smaller than the implant sizer was chosen.

We believe that at least 3 important points must be considered when trying to obtain clinically significant aesthetic outcomes in prepectoral direct-to-implant breast reconstruction using the ADM-assisted technique.

First, accurate knowledge of the surgical procedure and the selection of optimally sized silicone implants are crucial to obtain postoperative breast volume symmetry. Several studies have reported the clinical outcomes of prepectoral direct-to-implant breast reconstruction using the ADM-assisted technique, but none have reported on the selection of the silicone implant size. Therefore, the present study contributes to the reporting of postoperative breast volume symmetry in patients who have undergone prepectoral direct-to-implant breast reconstruction via the ADM-assisted technique, showing that selecting a smaller-than-planned silicone implant size provides superior results. We emphasize this because, in the aesthetic planning of breast reconstruction, the size of the silicone implant is typically chosen intraoperatively using an implant sizer. This is because the silicone implant must be selected before it is covered with the ADM. Once the silicone implant has been placed in the surgical field, it is difficult and expensive to change. Therefore, a careful approach is required to select the optimal silicone implant size. We also carried out procedures using a silicone implant that was the same size as the sizer and thought we had achieved volume symmetry. However, there was an increase in the number of complications of postoperative breast volume asymmetry found on follow-up examination in these cases.

Second, appropriate patient selection based on the indications for the procedure is essential to ensure the success of breast reconstruction. We only included patients with no contraindications to prepectoral implant-based breast reconstruction, in accordance with the Joint Guidelines of the Association of Breast Surgeons and the British Association of Plastic, Reconstruction, and Aesthetic Surgeons.^[14]^ Moreover, we excluded patients with conditions that might negatively impact the skin flap, such as preoperative radiotherapy history, current smoking status, and uncontrollable diabetes mellitus. In addition, we excluded patients with severely ptotic breast because, in such patients, blood supply to the postmastectomy skin flap is generally poor, and there is a high risk of implant malposition during healing within the large implant pocket. By performing strict patient selection, we excluded other biases and could investigate the factors influencing optimal silicone implant selection in prepectoral direct-to-implant breast reconstruction using the ADM-assisted technique.

Third, appropriate wrapping of the ADM cover plays an important role in prepectoral direct-to-implant breast reconstruction using the ADM-assisted technique. With improvements in NSM techniques, prepectoral direct-to-implant breast reconstruction has recently become more common. Debate remains regarding how to cover the implant with ADMs for prepectoral implantation. In dual-plane reconstruction, the ADM is mainly used to cover the lower pole of the implant. However, the ADM is used slightly differently in prepectoral direct-to-implant breast reconstruction. When placing an implant in the prepectoral plane, the ADM can cover either only the anterior surface of an implant or the entire surface.^[2]^ Complete coverage of breast implants with ADM reduces inflammation and prevents capsular contracture.^[17]^ Cases of capsular contracture after dual-plane reconstruction have been treated using complete implant coverage with ADM.^[18]^ Wrapping an implant with ADM prevents implant malposition, minimizes upper pole surface irregularities, and allows for natural-looking, soft breasts without detachment of the pectoralis major muscle. In the present study, the ADM was wrapped around the implant in our own manner to achieve a completely ADM-covered implant pocket.

Although several studies have reported on the clinical outcomes of prepectoral breast reconstruction, most of them focused on patient selection, surgical techniques, ADM coverage design, and postoperative care.^[17–19]^ Few studies directly referred to the importance of implant selection. Therefore, this study, with a large sample size, highlights the importance of predicting the volume of the ADM when selecting a silicone implant to achieve better postoperative breast volume symmetry.

Many plastic surgeons have only recently included complete coverage with ADM in breast reconstruction due to the high cost of the material. Therefore, it is easy to overlook the volume effects of ADM itself because it is merely considered a paper-like material that covers the implant to lower the risk of capsular contracture. Table 4 shows that postoperative breast volume symmetry was highest when an implant 50 cc smaller than the implant sizer was selected. These results indicate that ADM has a significant effect on the volume of the breast in the prepectoral direct-to-implant breast reconstruction using the ADM-assisted technique. Indeed, among many variables, the ADM size had a significant effect on the selection of the silicone implant size (Table 2).

This study had some limitations. Firstly, the size of the ADM used to cover the implant differed depending on whether large or small silicone implants were used during breast reconstruction. Additional research is needed to determine whether it is best to uniformly use a silicone implant that is 50 cc smaller than the implant sizer. Secondly, the follow-up period for measuring the symmetry score of AIS was limited. Despite these limitations, this study discussed silicone implant size selection, which is easy to overlook amongst the many important considerations needed to maximize the aesthetic outcome in prepectoral direct-to-implant breast reconstruction using the ADM-assisted technique. We found that the volume of the ADM influenced the selection of the breast implant size, and that it is best to perform prepectoral direct-to-implant breast reconstruction by selecting a silicone implant that is 50 cc smaller than the implant sizer.

## Author contributions

**Conceptualization:** Hyo Young Kim, Hong Il Kim, Hyung Suk Yi, Jin Hyung Park.

**Data curation:** Ho Sung Kim, Seok Kyung In, Byeong Seok Kim.

**Formal analysis:** Yoon Soo Kim, Ho Sung Kim, Seok Kyung In, Byeong Seok Kim.

**Methodology:** Seok Kyung In.

**Project administration:** Ho Sung Kim, Byeong Seok Kim, Jin Hyung Park.

**Software:** Ho Sung Kim.

**Supervision:** Jin Hyung Park.

**Visualization:** Ho Sung Kim, Hyo Young Kim.

**Writing – original draft:** Yoon Soo Kim.

**Writing – review & editing:** Yoon Soo Kim, Ho Sung Kim, Hyo Young Kim, Hong Il Kim, Hyung Suk Yi, Jin Hyung Park.

## References

[1] Storm-Dickerson T Sigalove N. Prepectoral breast reconstruction: the breast surgeon’s perspective. Plast Reconstr Surg. 2017;140(Prepectoral Breast Reconstruction):43S–8S. 29166347 10.1097/PRS.0000000000004050

[2] Ter Louw RP Nahabedian MY. Prepectoral breast reconstruction. Plast Reconstr Surg. 2017;140(Advances in Breast Reconstruction):51S–9S. 29064922 10.1097/PRS.0000000000003942

[3] Pacella SJ. Evolution in tissue expander design for breast reconstruction: technological innovation to optimize patient outcomes. Plast Reconstr Surg. 2018;142(The Science of Breast Implants):21S–30S. 10.1097/PRS.0000000000004999 30252756

[4] Colwell AS Damjanovic B Zahedi B. Retrospective review of 331 consecutive immediate single-stage implant reconstructions with acellular dermal matrix: indications, complications, trends, and costs. Plast Reconstr Surg. 2011;128:1170–8. 22094736 10.1097/PRS.0b013e318230c2f6

[5] McCarthy CM Mehrara BJ Riedel E. Predicting complications following expander/implant breast reconstruction: an outcomes analysis based on preoperative clinical risk. Plast Reconstr Surg. 2008;121:1886–92. 18520873 10.1097/PRS.0b013e31817151c4

[6] Srinivasa DR Garvey PB Qi J. Direct-to-implant versus two-stage tissue expander/implant reconstruction: 2-year risks and patient-reported outcomes from a prospective, multicenter study. Plast Reconstr Surg. 2017;140:869–77. 29068918 10.1097/PRS.0000000000003748 PMC5902733

[7] Rehnke RD Groening RM Van Buskirk ER. Anatomy of the superficial fascia system of the breast: a comprehensive theory of breast fascial anatomy. Plast Reconstr Surg. 2018;142:1135–44. 30511967 10.1097/PRS.0000000000004948 PMC6211786

[8] Sbitany H. Important considerations for performing prepectoral breast reconstruction. Plast Reconstr Surg. 2017;140(6S Prepectoral Breast Reconstruction):7S–13S. 10.1097/PRS.0000000000004045 29166342

[9] Nahabedian MY. Acellular dermal matrices in primary breast reconstruction: principles, concepts, and indications. Plast Reconstr Surg. 2012;130(Suppl 2):44S–53S. 23096984 10.1097/PRS.0b013e31825f2215

[10] Basu CB Leong M Hicks MJ. Acellular cadaveric dermis decreases the inflammatory response in capsule formation in reconstructive breast surgery. Plast Reconstr Surg. 2010;126:1842–7. 21124125 10.1097/PRS.0b013e3181f44674

[11] Jones G Yoo A King V. Prepectoral immediate direct-to-implant breast reconstruction with anterior AlloDerm coverage. Plast Reconstr Surg. 2017;140:31S–8S. 29166345 10.1097/PRS.0000000000004048

[12] Downs RK Hdges K. An alternative technique for immediate direct-to-implant breast reconstruction—a case series. Plast Reconstr Surg Glob Open. 2016;4:e821. 27536500 10.1097/GOX.0000000000000839 PMC4977149

[13] Cattelani L Polotto S Arcuri MF. One-step prepectoral breast reconstruction with dermal matrix-covered implant compared to submuscular implantation: functional and const evaluation. Clin Breast Cancer. 2018;18:e703–11. 29275104 10.1016/j.clbc.2017.11.015

[14] Visser NJ Damen THC Timman R. Surgical results, aesthetic outcome, and patient satisfaction after microsurgical autologous breast reconstruction following failed implant reconstruction. Plast Reconstr Surg. 2010;126:26–36. 20595835 10.1097/PRS.0b013e3181da87a6

[15] Martin L O’Donoghue JM Horgan K. Acellular dermal matrix (ADM) assisted breast reconstruction procedures: joint guidelines from the Association of Breast Surgery and the British Association of Plastic, Reconstructive and Aesthetic Surgeons. Eur J Surg Oncol. 2013;39:425–9. 23321393 10.1016/j.ejso.2012.12.012

[16] Dikmans REG Nene LEH Bouman MB. The aesthetic items scale: a tool for the evaluation of aesthetic outcome after breast reconstruction. Plast Reconstr Surg - Glob Open. 2017;5. 10.1097/GOX.0000000000001254 PMC5404439 28458968

[17] Schmitz M Bertram M Kneser U. Experimental total wrapping of breast implants with acellular dermal matrix: a preventive tool against capsular contracture in breast surgery? J Plast Reconstr Aesthet Surg. 2013;66:1382–9. 23764323 10.1016/j.bjps.2013.05.020

[18] Cheng A Lakhiani C Saint-Cyr M. Treatment of capsular contracture using complete implant coverage by acellular dermal matrix: a novel technique. Plast Reconstr Surg. 2013;132:519–29. 23985627 10.1097/PRS.0b013e31829acc1e

[19] Nealon KP Weitzman RE Sobti N. Prepectoral direct-to-implant breast reconstruction: safety outcome endpoints and delineation of risk factors. Plast Reconstr Surg. 2020;145:898e–908e. 32332523 10.1097/PRS.0000000000006721

